# Disaster literacy in disaster emergency response: a national qualitative study among nurses

**DOI:** 10.1186/s12912-024-01911-2

**Published:** 2024-04-24

**Authors:** Di Zhang, Li-Yan Zhang, Ke Zhang, Han Zhang, Huan-fang Zhang, Kai Zhao

**Affiliations:** 1 International Academy of Red Cross, Soochow University, Lvbo Lou, 1 Shizi Street, Jiangsu, China; 2 Disaster Nursing Committee, Chinese Nursing Association, Beijing, China; 3 School of Health Management, China Medical University, Shenyang, China; 4 The First Affiliated Hospital of University of South China, Hengyang, Hunan China; 5 The Third Affiliated Hospital of Guangzhou Medical University, Guangdong, China; 6 The Affiliated Hospital of Xuzhou Medical University, Jiangsu, China

**Keywords:** Disaster literacy, Emergency management, Nurse, Qualitative research

## Abstract

**Background:**

As the largest group of healthcare professionals, nurses play an indispensable and crucial role in disaster response. The enhancement of nurses’ disaster literacy is imperative for effective disaster emergency management. However, there is currently a lack of knowledge regarding nurses’ disaster literacy. This study represents the first attempt to explore the key components and characteristics of disaster literacy among nurses.

**Methods:**

A qualitative descriptive design was employed, and the reporting followed the Consolidated Criteria for Reporting Qualitative Research (COREQ) guidelines (refer to File S1). The purposive sampling method was utilized. Thirty-one rescue nurses from 31 medical institutions across 25 provinces and regions in China were recruited to participate in the study. The respondents were requested to share their experiences and insights regarding disaster rescue operations. Inductive content analysis was employed for data examination.

**Results:**

The results indicated that rescue nurses universally recognized that there was a pressing need to enhance the level of disaster literacy among nurses. The disaster literacy of nurses encompasses nine dimensions: physical and mental quality, disaster rescue general knowledge, professional and technical competence, professional ethics, teamwork, emotional ability, information literacy, leadership, and knowledge transformation.

**Conclusions:**

To ensure national sustainability, government departments, healthcare organizations, and hospital administrators can accurately evaluate the disaster literacy of individual clinical nurses, groups, and the workforce as a whole through nine dimensions, which also can provide evidence to support the development of precision strategies to strengthen the disaster literacy of nurses.

**Supplementary Information:**

The online version contains supplementary material available at 10.1186/s12912-024-01911-2.

## Background

Disaster prevention has long been a priority of the international healthcare system [1]. Effective disaster risk management is intricately intertwined with the attainment of the Global Sustainable Development Goals [2]. In 2020, for instance, the unforeseen proliferation of COVID-19 impeded global endeavors to accomplish the Sustainable Development Goals worldwide [3]. Nevertheless, this pandemic has also engendered an unparalleled surge in media and public attention towards nursing [4], along with a global discourse regarding the pivotal role played by nursing professionals in national disaster response and public safety.

Nurses are the largest group of healthcare professionals globally and serve as the primary responders during disaster rescue operations [5]. Enhancing disaster literacy within this extensive group can significantly enhance treatment efficiency, an aspect that is often overlooked [6]. Disasters frequently occur unexpectedly, resulting in chaotic and intricate environments at rescue sites, which starkly contrasts with structured clinical settings comprising fixed clinical departments and medical staff. Consequently, experienced or trained nurses are indispensable for managing the complexities associated with such situations. The demanding treatment environment characterized by inadequate resources, scarcity of supplies and medications, urgent tasks, and psychological stress places heightened demands on nurses engaged in disaster rescue efforts. According to the State of the World’s Nursing Report 2020, advancing disaster nursing disciplines will play a pivotal role in shaping the future of global nursing [7]. Consequently, adapting traditional first aid structures, nursing skills, and theoretical frameworks to suit disaster scenarios has proven challenging.

Despite this, not every nurse is adequately prepared to confront these situations. Studies have confirmed that nurses’ knowledge, skills, and abilities in disaster emergency response are insufficient to deal with such circumstances [8–9]. Furthermore, it should be noted that the concepts of knowledge, skill, ability, and literacy are distinct and cannot be equated. In the context of disaster rescue operations, saving lives is a crucial ability, life first is the value of faith, and dedication is the necessary character. The disaster rescue scene serves as a litmus test for nurses’ emergency response capabilities, however, possessing the necessary ability does not necessarily imply having literacy in this domain. According to the Organisation for Economic Co-operation and Development (OECD), literacy encompasses not only knowledge and skills but also the capacity to utilize psychosocial resources effectively in complex situations [10]. Literacy possesses multiple dimensions, extending beyond the mere acquisition of knowledge or skills, with a greater emphasis on their practical application for problem-solving during critical incidents. Consequently, disaster literacy aims at enhancing nurses’ disaster nursing abilities by integrating and promoting their knowledge and skills, processes and methods, emotional attitudes, as well as values [11]. The development of disaster literacy proves advantageous for nurses when confronted with diverse emergencies that arise from local conditions within a disaster scenario.

However, there is currently a lack of global studies examining the conceptual connotation of disaster literacy in professional groups, specifically focusing on nurses. Furthermore, existing research indicates that nurses may be inadequately prepared to effectively respond to disasters [12–13]. Therefore, it is imperative to discuss the significance of disaster literacy among nurses and elucidate the characteristics that nurses should possess.

## Methods

### Design

The qualitative and descriptive design was used in this study to determine the disaster literacy characteristics that nurses should have from the perspective of rescue nurses. The present study employed a qualitative and descriptive design to ascertain the disaster literacy characteristics that nurses should possess from the perspective of rescue nurses. Utilizing a qualitative descriptive design is considered the most effective approach for directly gathering information from survey participants [14]. Qualitative descriptive research aims to provide comprehensive accounts of experiences in authentic settings using straightforward language, thereby enabling detailed descriptions of otherwise unknown occurrences [15]. Consequently, employing a qualitative descriptive study design ensures that data analysis remains faithful to participants’ descriptions and facilitates transparency in researchers’ judgments.

To ensure the interviews remain focused on the topic, this study developed an interview outline based on the STAR tool [16]. The STAR method is a commonly employed technique by interviewees to effectively respond to behavioral interview questions. STAR stands for Situation, Task, Action, and Result. Through utilizing the STAR method, interviewees articulate their job-related scenarios eloquently, describe their responses comprehensively, and provide detailed outcomes when addressing behavior-based inquiries. Thus, the utilization of the STAR method guarantees that interviewees are capable of delivering concise and persuasive answers. The formal interview outline is finalized after thorough review and modification by experts in the qualitative research field within the project team as presented in Table 1.

**Table 1:** The STAR path of interview guide

Interview path	Interview outline
S(Situation)	What kind of situation did you find yourself in? What caused or contributed to the situation? Who were the individuals and organizations involved in this matter? What kind of role did you play?
T(Task)	What were the most important tasks you had to complete in that situation? What was the objective that had to be met?
A(Action)	What were your true feelings and thoughts in that situation? What steps were you willing to take? What did exactly you do and say? Why did you think and act that way? What did you think of the other people who were present? (particularly emotional, firsthand knowledge of events, and emotional feelings)
R(Result)	What was the outcome? What was your experience and how did you feel afterward? What was the most important factor in your success, in your opinion?

### Setting and participants

The purposive sampling method was employed to recruit participants. The selection criteria for participants are as follows: (1) Licensed registered nurses from medical and healthcare institutions nationwide; (2) Participation in at least two out of the four major disaster types, including natural disasters (earthquakes, floods, snowstorms, etc.), accident disaster (mining accidents, traffic accidents, accidents at public facilities and equipment, etc.), public health events (SARS, Avian Influenza, New Crown Pneumonia Pandemic, etc.), and social security events (mass incidents, terrorist attacks, emergencies affecting market stability, particularly significant foreign-related incidents, etc.); (3) Willingness to be interviewed. Recruitment and selection encompass China’s seven administrative geographic regions. The exclusion criteria are as follows: (1) Involvement in fewer than two disaster rescue events; (2) Incomplete provision of personal and disaster relief information during formal interviews leading to unanalyzable data; (3) Insufficient interview time available for the participant.

### Data collection

The recruitment notice was disseminated to all provincial disaster nursing professional committees through the Disaster Nursing Professional Committee of the Chinese Nursing Association in this study. Rescue nurses who met the inclusion criteria were contacted via email or phone as stated in the recruitment notice, and they provided us with their personal contact information. Before conducting pre-interviews and formal interviews, we communicated with each individual by phone or email and sent them an informed consent form, which they signed and returned to our research team. The qualitative descriptive design was employed to collect individual interview data from July to September 2021. Due to the influence of epidemic prevention and control measures during that period, telephone interviews were conducted for this study. Before the formal interviews, two participants were selected by the interview team for pre-interviews to identify and address any issues that may arise during the interviews, ensuring their effectiveness. The formal interviews commenced by adhering to the interview outline and requirements. The researchers meticulously recorded each interview, capturing nuances such as tone of voice, intonation, pauses, etc., and appropriately labeling them. Each interview was transcribed verbatim upon initial recording. The duration of these interviews ranged from 30 to 77 min with an average of 47 min.

### Data analysis

This report adheres to the EQUATOR Guidelines for Research Reporting as well as the Comprehensive Criteria for Reporting Qualitative Research (COREQ), which comprises a set of 32 items suitable for individual interviews [17] (refer to File S1). In China, data collection and analysis were conducted concurrently. The data underwent six steps of inductive content analysis [18]. Each step was meticulously validated by the researchers to ensure the quality and credibility of the analysis [19]. Two researchers listened to the recordings several times, independently analyzed the texts, and subsequently engaged in discussions until reaching a consensus.

### Rigour

We employed the qualitative research methodology developed by Graneheim et al. to enhance the credibility, reliability, verifiability, transferability, and authenticity of our study [20]. During the recruitment phase, we established inclusion and exclusion criteria to identify participants with extensive experience in disaster rescue operations and their ability to effectively articulate those experiences. Considering that content analysis emphasizes variations in content, diversity, and differences across different types of disasters, we conducted purposive sampling to recruit participants from various regions across the country for interviews. To develop an interview outline, we sought guidance from esteemed experts in disaster response and qualitative research. Before the interviews, researchers underwent comprehensive training in systematic qualitative research and content analysis to acquire relevant knowledge and refine their interviewing skills. In order to fine-tune the interview questions during the process and establish a clear framework for the formal interviews, two pre-interviews were conducted by the interview team. Text analysis was performed independently by two researchers, followed by extensive discussions until a consensus was reached [21]. Moreover, this study employed a six-step method of qualitative content analysis for category analysis to ensure credibility and authenticity [18]. Original recordings, transcripts, and coding memos from all participants were retained for auditing purposes as well as cross-verification. Additionally, adherence to the Comprehensive Criteria (COREQ) outlined in the report’s inventory of qualitative studies was strictly observed [17].

### Ethics approval and consent to participate

This study is part of a larger research project, and all phases of this study have received approval from the Medical Ethics Committee of Jiangsu University. All participants were provided with information regarding the purpose of the study, and both verbal and written informed consent was obtained after emphasizing that participation was entirely voluntary. Additionally, all participants were notified about the strict confidentiality measures in place for their interview data. They had the option to withdraw from the study at any time, with the assurance that their interview data would be deleted. Furthermore, all data has been anonymized and de-identified, with restricted access limited to researchers only.

## Results

A total of 33 disaster nurses were recruited for interviews in this study, but one could not be reached and one was interviewed for less than 30 min. Consequently, we obtained 31 valid interview materials. Nurse codes were assigned from N1 to N31, using the first letter of each nurse’s name as their code number. Descriptive information for each participant is provided in Table 2. The average age of the interviewed nurses was (38.45 ± 6.51) years old, with an average RN experience of (16.77 ± 7.50) years. On average, they had participated in disaster rescues (2.87 ± 0.92) times and belonged to a category of disaster rescue with an average rating of (1.87 ± 0.56). The interviews lasted on average for (47.23 ± 12.63) minutes. The demographics are listed in Table 3.

**Table 2:** Descriptive information of the participants (*n* = 31)

Nurses	Age	Gender	Geographical regions	RN experience(Years)	Number of disaster rescue	Category of disaster rescue	The length of the interviews(minute)
N1	34	Female	Northeast	16	2	1	42.58
N2	42	Female	Northeast	20	3	2	53.20
N3	41	Female	Northeast	20	3	2	32.58
N4	35	Male	East	12	2	2	52.72
N5	35	Male	South	10	2	2	62.17
N6	34	Male	South	12	6	1	57.97
N7	35	Male	North	15	3	2	63.35
N8	39	Female	East	16	2	2	34.37
N9	37	Male	East	14	2	2	47.98
N10	42	Female	East	17	2	2	31.15
N11	38	Female	East	19	2	2	54.75
N12	36	Male	East	15	4	1	41.33
N13	34	Female	East	10	2	1	30.58
N14	26	Male	Central	6	2	2	54.80
N15	36	Male	Central	12	4	2	63.82
N16	28	Male	Central	5	4	2	49.25
N17	39	Male	East	16	2	2	59.88
N18	48	Female	North	29	2	2	76.63
N19	33	Female	South	10	3	3	35.78
N20	31	Male	South	10	4	2	43.67
N21	32	Male	Southwest	7	3	3	37.37
N22	37	Female	North	18	3	1	35.77
N23	37	Male	Northwest	15	3	2	70.55
N24	45	Female	Northwest	24	3	3	35.82
N25	50	Female	Northwest	29	2	2	45.90
N26	55	Female	Southwest	36	3	1	41.57
N27	38	Female	Southwest	14	3	2	49.27
N28	51	Female	Southwest	33	3	2	32.30
N29	40	Female	Southwest	17	4	1	29.22
N30	44	Female	Southwest	22	3	2	55.87
N31	40	Female	Southwest	21	3	2	42.07

**Table 3:** Demographics of the participants (*n* = 31)

Characteristics Variable	Frequency(n)	Ratio(%)
**Region**		
Northeast	3	9.7
North	3	9.7
East	8	25.8
South	4	12.9
Central	3	9.7
Northwest	3	9.7
Southwest	7	22.6
**Gender**		
Female	18	58.1
Male	13	41.9
**Highest education attained**		
Undergraduate	27	87.1
Postgraduate	4	12.9
**Clinical department**		
Critical care medicine	4	12.9
Emergency	16	51.6
Pre-hospital emergencyt	3	9.7
Anesthesia surgery	3	9.7
Orthopedics	2	6.5
Cardiology	1	3.2
Vascular surgery Nucleic acid collection room	1 1	3.2 3.2
**Occupational title**		
No	13	41.9
Deputy head nurse	5	16.1
Head nurse	13	41.9
**Professional title**		
Nurse	2	6.5
Nurse in charge	15	48.4
Deputy chief nurse	13	41.9
Chief nurse	1	3.2
**Number of disaster rescue efforts**		
Twice	12	38.7
Three times	13	41.9
Four times or more	6	19.3
**Participate in disaster rscue categories**		
Natural disaster	14	45.2
Accident disaster	12	38.7
Public health event	24	77.4
Social security incident	7	22.6

All interviewed disaster rescue nurses unanimously agreed on the critical importance of studying disaster literacy in nursing and emphasized that government departments and healthcare institutions should promptly enhance education and training programs for nurses, including nursing students, to effectively respond to various potential disasters. Additionally, data from interviews were analyzed and summarized to extract nine essential characteristics of disaster literacy that nurses should possess, as presented in Table 4.

**Table 4:** Results of inductive content analysis

Category	Subcategory
Physical and mental quality	Have a high level of physical quality and stamina. Self-psychological adjustment can take place at various stages of disaster rescue. Be able to effectively manage emotions in the face of emergencies or stressful situations. Can provide psychological counseling and emotional support to disaster victims and rescue workers.
Disaster rescue general knowledge	Recognize the characteristics and nature of various types of disaster events. Be able to assess disaster situations in disaster zones (climate conditions, folk customs, religions, major disease spectrum, possible secondary disasters, etc.). Be able to use personal protective equipment and existing resources to keep oneself and others safe.
Professional and technical competence	Demonstrate comprehensive theoretical knowledge of the nursing profession. Familiar with common pre-hospital emergency nursing knowledge. Master common hospital emergency nursing operation skills. Master basic pre-hospital emergency nursing skills (cardiopulmonary resuscitation, trauma hemostasis, bandaging, fixation, handling, etc.).
Professional ethics	Possess certain political and ideological qualities, as well as a sense of confidentiality. Professional commitment and individual initiative. Professional belief, sense of responsibility, and belief that life is important. Understand and apply ethical practices in disaster response based on the utilitarian principle.
Teamwork	High medical cooperation ability. Have the ability to collaborate on a multidisciplinary or departmental level.
Emotional ability	Be able to communicate effectively with disaster victims/rescue workers. Empathic ability. Offering humanistic care during disaster rescue.
Information literacy	Maintain current emergency resources, plans, policies, and procedures. Be able to communicate/report priority disaster-related information to superior leaders or designated personnel promptly. Be able to communicate in multiple languages with disaster victims and rescue workers. Work with the disaster leadership team to develop media information about disaster events. Disaster site nursing execution that is quick and efficient.
Leadership	Capable of exercising certain organizational, coordination, and management skills (personnel, materials, information, environment, time, etc.). Develop on-site working procedures in accordance with emergency plans and specific situations. It can aid in the organization of rescue operations and ensure the work of vulnerable groups.
Knowledge transformation	Be able to criticize the application of knowledge and skills to deal with disaster site situations based on local conditions. Disaster information can be integrated for decision-making. Disaster health education and training can be carried out during and after disasters. Scientific research on disaster rescue can be conducted.

### Physical and mental quality

Most participants concurred that nurses must possess physical fitness and stamina to effectively carry out high-intensity rescue work in extreme environments at disaster sites. Nearly all participants agreed that nurses should have the ability to self-regulate and manage their emotions during the process of disaster response. Additionally, all participants emphasized the significance of nurses promptly recognizing and intervening when a patient or injured individual is undergoing a psychological crisis.

If your physical condition is not optimal, you may become a liability to the team when going out to rescue people. (N31).

He suffered a head injury during the earthquake, but as an infant, he was remarkably adorable with plump white cheeks resembling a small meatball… Later on, despite leaving that ward I never wanted to enter again, whenever I think of his chubby figure lying uncomfortably on the hospital bed, it reminds me of the profound psychological impact caused by this experience. (N2).

### Disaster rescue general knowledge

The majority of participants emphasized the importance for nurses to possess a comprehensive understanding of the characteristics, nature, and specific circumstances associated with different types of disaster events. Additionally, they should be equipped with general knowledge to effectively safeguard their safety as well as that of others at the disaster site.

After undergoing nearly two months of intensive training, primarily focused on epidemic prevention, isolation protocols, disease nursing techniques, and cultural sensitivities in Africa, as well as language instruction, we must acquire a comprehensive understanding of the highly contagious nature of Ebola before we can effectively assist. (N25).

The lack of preparedness was evident during our rescue mission, encompassing ourselves, the supplies we brought, and a significant portion of our management, personnel, and materials. This remains a regrettable oversight. (N20).

### Professional and technical competence

The participants unanimously emphasized the importance for nurses to possess a comprehensive understanding of theoretical knowledge in the field of nursing, proficient skills in hospital emergency nursing procedures, and fundamental abilities in on-site rescue nursing before engaging in rescue operations. Additionally, they stressed the necessity for nurses to be well-versed in common pre-hospital emergency nursing knowledge.

…. Subsequently, her blood pressure and vital signs exhibited a decline, necessitating an accelerated administration of fluids while adjusting the patient’s position to manage shock. I am grateful for the invaluable experience gained during my rotation in anesthesiology, particularly in mastering intubation techniques… (N28).

When transferring patients, our medical staff should possess not only exceptional professional skills but also a profound understanding of the characteristics of disaster rescue and potential accidents. (N30).

### Professional ethics

The majority of participants emphasized the importance for nurses to possess political acumen and maintain strict confidentiality during rescue operations. Additionally, they highlighted the significance of professional dedication, subjective initiative, prioritizing life above all else, and a strong sense of responsibility. Moreover, nurses need to comprehend and apply the utilitarian principle in their ethical practice.

Considering the potential hazards present at disaster sites, it is imperative to foster a spirit of sacrifice. It is crucial to harness the selfless dedication instilled in us during our medical education. For instance, during a pandemic like COVID-19, one must be willing to make personal sacrifices for survival. (N3).

The addition I propose is the cultivation of awe, a reverence for life, and unwavering faith. The fundamental purpose of studying medicine should remain unchanged - prioritizing the patient’s well-being and valuing human life above all else. This mindset is crucial as it embodies the spirit of selfless dedication. (N23).

### Teamwork

The overwhelming majority of participants emphasized the paramount importance of nurses possessing a robust capacity for medical/nursing collaboration, as well as adeptness in multi-/cross-disciplinary or sectoral integration within rescue scenarios.

The second point is that in the event of an emergency, it is crucial to promptly assemble a highly proficient medical team. (N17).

The field of rescue operations necessitates not only intra-unit collaboration but also inter-unit cooperation, thereby demanding specific interdisciplinary collaborative skills. (N24).

### Emotional ability

The consensus among all participants was that nurses should possess the ability to effectively communicate with both disaster victims and rescue workers, particularly through perspective-taking and empathy. Furthermore, they should be capable of providing humanistic care during disaster rescue operations.

Communication with disaster victims is also crucial as it plays a vital role in alleviating their fears and facilitating their acceptance of treatment, thus promoting cooperation during the treatment process. (N25).

Many patients are experiencing extreme fear and anxiety due to their lack of knowledge about the novel coronavirus, leading them to believe that mere contact with it will result in death. While some patients remain silent, others display visible signs of concern… It is crucial to address the emotional distress experienced by different patients and employ empathy as a means to alleviate their negative emotions. (N9).

### Information literacy

The majority of participants emphasized the importance for nurses to possess updated knowledge of rescue resources, emergency plans, policies, and procedures. Additionally, they should be proficient in promptly communicating/reporting priority disaster-related information to superiors or designated personnel at the disaster site and efficiently implementing it. Moreover, fluency in multiple languages is essential for effective communication with both disaster victims and rescue workers. Furthermore, collaboration with disaster leadership teams to develop media information on disaster events is also crucial.

The initial step we took was to break through the English language barrier by identifying and familiarizing ourselves with frequently used sentences and keywords in our processing. Subsequently, we engaged in effective communication with their leaders and translators. (N13).

Many departments will request information, including the municipal government, the Health and Construction Commission, the emergency office, and some leaders in charge. How should this information be saved? Who has access to this information? We’d been… I was completely perplexed when I arrived on the scene. (N4).

### Leadership

The majority of participants emphasized the necessity for nurses to possess the ability to contribute to the organization of disaster emergency plans or the development of on-site work processes, as well as demonstrate proficient organizational coordination and management skills while assisting in safeguarding vulnerable populations.

The individual should exhibit decisiveness, demonstrate effective leadership skills, possess contingency planning abilities, and discern between right and wrong actions while articulating the rationale behind choosing the correct course of action. (N9).

I believe it is crucial to adopt a disaster management mindset.… There should be an enhancement in the thinking and capabilities related to managing sudden major incidents. (N19).

### Knowledge transformation

The significance of nurses’ ability to effectively respond to disasters and integrate disaster information into decision-making was unanimously emphasized by all participants. Concurrently, disaster health education, training, and scientific research can be conducted both during and after the occurrence of a disaster.

As a disaster rescue worker, it is essential to possess adaptability in response to unforeseeable circumstances. This entails the ability to modify work procedures and status based on real-time changes. (N25).

Unfortunately, we don’t have a lot of time to research the front lines, which is something that must be done. (N11).

## Discussion

The findings of this study revealed that all participants provided positive feedback regarding the implementation of the disaster literacy study for nurses, as evidenced by the analysis of interview results. They urgently call upon relevant authorities, such as government bodies or healthcare organizations, to prioritize disaster literacy education and training for nurses. Furthermore, through feedback analysis, nine essential characteristics of disaster literacy that nurses should possess have been identified. To our knowledge, this is the first study to establish a comprehensive definition of disaster literacy specifically tailored for nurses. Consequently, it proposed a specific connotation of nurses’ disaster literacy based on their practical rescue experience and significantly contributes to the theoretical development framework within the field of disaster response.

The STAR path was employed in this study to facilitate one-on-one interviews, addressing the time constraints faced by nurses and enabling them to gather data effectively. This approach aided in refining the interview format, guiding participants’ attention toward event details, and encouraging reflection on specific experiences of interest. Thus, interviewers were able to delve into theories and establish characteristic indicators during the interviewing process. As a result, it is recommended that this methodology be widely adopted in future qualitative research endeavors.

The findings of this study are consistent with previous research [6, 22–24], which indicates that nurses should prioritize their attention on dimensions such as physical and mental well-being, professional and technical competence, teamwork skills, and emotional resilience in disaster literacy. The majority of the participants in this study reported experiencing chaotic conditions at the disaster scene, encountering challenging tasks, being in suboptimal physical condition, and often being unable to complete rescue missions. Certain natural disasters and accidents tend to occur in harsh environments or regions. Nurses working in high-stress environments must possess robust emotional intelligence, adaptability to dynamic situations, and strong collaborative skills for effective task completion during extreme circumstances. Participants generally agreed that nurses should be capable of psychological and emotional adjustment throughout the process of disaster rescue, while also providing psychological counseling and emotional support to both victims and rescue personnel. As a result, effective psychological first-aid training is critical [25–26]. According to a recent systematic review, cognitive behavioral therapy, psychoeducation, or meditation may assist nurses in overcoming their lack of emotional preparedness [27]. Furthermore, professional preparedness is essential for effective disaster response. Existing research, however, indicates that nurses are not fully prepared for a disaster emergency [28]. According to studies, themed game-based training is more effective than traditional scenario simulation and case teaching in improving nurses’ disaster response-ability [29–30]. As a result, managers should establish corresponding disaster training programs to enhance nurses’ disaster literacy.

Another significant finding of this study is that disaster rescue general knowledge, professional ethics, and information literacy are considered crucial dimensions of nurses’ disaster literacy. This aspect has been rarely reported in previous studies, thereby enhancing the development framework of disaster rescue disciplines. Because disaster events occur unexpectedly, nurses must respond effectively and quickly, so nurses must understand the various types and characteristics of disasters. Acquiring such rescue general knowledge will enable nurses to enhance their self-awareness regarding disasters and facilitate quicker and more efficient responses [31]. Participants in this study emphasized that nurses involved in disaster rescue should possess not only initiative and professionalism but also a profound professional conviction regarding the value of life and a genuine passion for nursing work. This ensures that nurses are willing to provide essential care during such catastrophic events. Moreover, it is crucial to address the severe global nurse shortage [32], inadequate nursing manpower, and inefficient disaster response systems [33]. Previous research has demonstrated that organizational support plays a pivotal role in enhancing nurses’ engagement and reinforcing their professional beliefs [34]. As a result, managers should utilize this data to enhance disaster management policies, increase nursing manpower, and encourage more nurses to volunteer for disaster rescue work. It is worth noting that some study participants mentioned that nurses should have important qualities such as timely follow-up on disaster information, timely reporting of priority information at the disaster site, and if permitted, joint development of media information with managers. Although rarely discussed, these aspects will be the focal point of future research on disaster nursing.

According to this study, enhancing leadership and knowledge transfer skills serves as a crucial indicator of nurses’ disaster literacy, which is reflected in organizational coordination, developing procedures or processes, safeguarding vulnerable groups, critical thinking, integrating information, health education, and scientific research. A disaster is defined as an occurrence that disrupts the normal functioning of a community and requires the utilization of external human and/or material resources for assistance [35]. Currently, the role of nurses in disaster rescue is not widely debated. Previous studies conducted by Chinese scholars have underscored that nurses’ involvement in disaster emergencies goes beyond being mere clinical nursing providers and encompasses crucial responsibilities such as coordination, problem-solving, and education [36]. This study confirms these findings. Disaster nursing significantly differs from clinical nursing as it necessitates nurses to handle a myriad of complex emergencies based on local circumstances. As a result, it is critical to strengthen the training of nurses’ transformational learning through daily disaster education and training, which will also be a focal point for future development in the field of disaster nursing discipline and the cultivation of nurses’ disaster literacy.

## Limitations

The study was conducted within the same country. The qualitative approach could reflect the picture of disaster literacy from the Chinese context. However, it is important to note that certain in-depth information may only be applicable in specific contexts due to variations in the frequency and magnitude of different types of disasters. Furthermore, given that this study took place during the COVID-19 pandemic in China, caution should be exercised when generalizing the results across all phases of disasters.

## Conclusions

This study identifies nine dimensions of disaster literacy that nurses should possess from the perspective of disaster rescue nurses. Including Physical and mental quality, Disaster rescue general knowledge, Professional and technical competence, Professional ethics, Teamwork, Emotional ability, Information literacy, Leadership, and Knowledge transformation. Disaster literacy research and practice among nurses must be promoted urgently by government agencies and medical institutions. Managers can utilize this feedback to enhance disaster management policies and provide continuous support for nursing professionals in their disaster response efforts through education, training, and effective management.

### Electronic supplementary material

Below is the link to the electronic supplementary material.

Supplementary Material 1

## Data Availability

The authors confirm that the data supporting the findings of this study are available within the article [and/or its supplementary materials]. All data included in this study are also available by contact with the corresponding author.
