# Supplementary material for: Disaster literacy in disaster emergency response: a national qualitative study among nurses
Source: BMC Nurs. 2024 Apr 24;23:267. doi: 10.1186/s12912-024-01911-2 (PMC11040771; doi:10.1186/s12912-024-01911-2)
Supplement: Supplementary file 1 — Supplementary Material 1 [file 12912_2024_1911_MOESM1_ESM.doc]

Table 1 Consolidated criteria for reporting qualitative studies (COREQ): 32-item checklist

| No Item | Guide questions/description |
| --- | --- |
| **Domain 1: Research team and reflexivity** | |
| Personal Characteristics | |
| 1．Interviewer/facilitator | *Di Zhang* |
| 2．Credentials | *PhD* |
| 3．Occupation | *Registered nurse* |
| 4．Gender | *Female* |
| 5．Experience and training | *Trained in qualitative research and disaster nursing* |
| Relationship with participants | |
| 6．Relationship established | *Yes* |
| 7．Participant knowledge of the interviewer | *They do not know each other, but they hope to contribute to the development of disaster nursing.* |
| 8．Interviewer characteristics | *The goal of the researcher is to provide an account of the “experiences, events and process that most people (researchers and participants) would agree are accurate”* |
| **Domain 2: study design** | |
| Theoretical framework | |
| 9．Methodological orientation and Theory | *Qualitative description approach , content analsis* |
| Participant selection | |
| 10．Sampling | *Purposive sampling* |
| 11．Method of approach | *Telephone* |
| 12．Sample size | *Thirty one* |
| 13．Non-participation Setting | 0 |
| 14．Setting of data collection | *Workplace* |
| 15．Presence of non-participants | *No* |
| 16．Description of sample | *The selection criteria for participants are as follows: 1) Licensed registered nurses from medical and health institutions across the country; 2) Taking part in at least two of the four major disaster types: natural disaster, accident disaster, public health event and social security event; 3) Volunteer to be interviewed.The interviews were conducted by telephone between July and September 2021.* |
| Data collection | |
| 17．Interview guide | *Yes, two pre-interviews were conducted and three experts were invited to optimize.* |
| 18．Repeat interviews | *Two pre-interviews and 32 formal interviews were conducted, with no repeat interviews.* |
| 19．Audio/visual recording | *Audio* |
| 20．Field notes | *Yes* |
| 21．Duration | *Between 30 and 77 minutes, with an average duration of 47 minutes.* |
| 22．Data saturation | *Yes* |
| 23．Transcripts returned | *Yes* |
| **Domain 3: analysis and findingsz** | |
| Data analysis | |
| 24．Number of data coders | *Two* |
| 25．Description of the coding tree | *Yes* |
| 26．Derivation of themes | *Derived from the data.* |
| 27．Software | *None* |
| 28．Participant checking | *Yes* |
| Reporting | |
| 29．Quotations presented | *Yes* |
| 30．Data and findings consistent | *Yes* |
| 31．Clarity of major themes | *Yes* |
| 32．Clarity of minor themes | *Yes* |
